# Searching for Programme theories for a realist evaluation: a case study comparing an academic database search and a simple Google search

**DOI:** 10.1186/s12874-020-01084-x

**Published:** 2020-08-26

**Authors:** Susanne Coleman, Judy M. Wright, Jane Nixon, Lisette Schoonhoven, Maureen Twiddy, Joanne Greenhalgh

**Affiliations:** 1 Leeds Institute of Clinical Trials Research, University of Leeds, Leeds, LS2 9JT UK; 2 University Medical Center Heidelberglaan 100, 3584 CX, Utrecht. Internal mail Str. 6.131 PO Box 85500, 3508 Utrecht, GA Netherlands; 3 Mixed Methods Research. Hull York Medical School, University of Hull, Cottingham Road, Hull, HU6 7RX UK; 4 Department of Sociology and Social Policy, University of Leeds, Leeds, LS2 9JT UK

**Keywords:** Realist evaluation, Programme theory, Scoping review, Literature searching, Information retrieval, Internet

## Abstract

**Background:**

Realist methodologies are increasingly being used to evaluate complex interventions in health and social care. Programme theory (ideas and assumptions of how a particular intervention works) development is the first step in a realist evaluation or a realist synthesis, with literature reviews providing important evidence to support this. Deciding how to search for programme theories is challenging and there is limited guidance available. Using an example of identifying programme theories for a realist evaluation of Pressure Ulcer Risk Assessment Instruments in clinical practice, the authors explore and compare several different approaches to literature searching and highlight important methodological considerations for those embarking on a programme theory review.

**Methods:**

We compared the performance of an academic database search with a simple Google search and developed an optimised search strategy for the identification primary references (i.e. documents providing the clearest examples of programme theories) associated with the use of Pressure Ulcer Risk Assessment Instruments (PU-RAIs). We identified the number of primary references and the total number of references retrieved per source. We then calculated the number needed to read (NNR) expressed as the total number of titles and abstracts screened to identify one relevant reference from each source.

**Results:**

The academic database search (comprising CINAHL, The Cochrane Library, EMBASE, HMIC, Medline) identified 2 /10 primary references with a NNR of 1395.The Google search identified 7/10 primary references with a NNR of 10.1. The combined NNR was 286.3. The optimised search combining Google and CINAHL identified 10/10 primary references with a NNR of 40.2.

**Conclusion:**

The striking difference between the efficiency of the review’s academic database and Google searches in finding relevant references prompted an in-depth comparison of the two types of search. The findings indicate the importance of including grey literature sources such as Google in this particular programme theory search, while acknowledging the need for transparency of methods. Further research is needed to facilitate improved guidance for programme theory searches to enhance practice in the realist field and to save researcher time and therefore resource.

## Background

Realist methodology is increasingly used in the evaluation of complex health care interventions, to facilitate a deeper understanding of ‘what works, how, for whom, in what circumstances and to what extent’ [1–4]. The initial step of realist studies aims to identify programme theories underlying the intervention . A programme theory is an explanation of how and why an intervention is expected to work and is often expressed as a Context Mechanism Outcome (CMO) configuration. Programme theory identification is an iterative process and can involve a range of methods including reviewing existing literature, documentation review, interviews with stakeholders, focus groups and/or reviewing relevant theories from other literature [2, 3, 5].

Ideas about programme theories can be found in a range of sources and are not limited to academic literature; they can be found in websites, blogs, newspaper articles, letters or even radio programmes [6]. Deciding how to search for programme theories is challenging as there is limited guidance for realist searches [3, 7, 8] compared with systematic reviews [9–12]. While both searches employ a wide range of strategies, searches for programme theories focus on identifying a different type of source compared to systematic reviews. This stems from the different underlying aims of the reviews. Systematic reviews employing a conventional ‘Cochrane style’ methodology to answer questions of treatment/diagnostic effectiveness, aim to identify all relevant empirical studies and often reflect strict inclusion/exclusion criteria e.g. for study design, outcomes, populations and settings. Multiple database searches and supplementary search techniques are recommended to reduce publication bias [11–14]. However, searches to support the identification of programme theories are concerned with identifying the full range of ideas and assumptions that underpin how the intervention is intended to work (rather than every paper containing a programme theory). These ideas often pertain to assertions about how the programme might work, the populations or situations in which the programme might work as intended and those where it might be expected not to work. Identifying these ideas often requires consideration of wider literature such as opinion papers, guidelines, blogs and organisation reports [8, 15] which may not be indexed in academic databases [16, 17]. Furthermore, at this stage of any realist enquiry, the focus is on identifying *plausible* ideas, rather than evidence to underpin the validity of those ideas; the testing and refinement of programme theory (by juxataposing these ideas with evidence) occurs in the next stage of any realist enquiry. As such, the sources used to identify programme theory are not subject to specific study design considerations.

Designing a search with a high recall (sensitivity) and high precision is challenge for both a review of theories and systematic reviews. Searches are designed with the aim of finding all relevant papers, while minimising retrieval of irrelevant papers. At the same time the searches must be robust and transparent [13, 18]. The most efficient way to achieve this for programme theory reviews remains unclear and many approaches are considered relevant, incorporating browsing library shelves, discussion with experts, citation tracking, web searching and cluster searching [8, 16, 17, 19–21]. Google is considered a quick and easy way to search for grey literature and other sources of programme theories (eg websites, blogs, newspaper articles etc). However, it is not considered a first choice of resource for systematic searching due to the impact of personalised search history and lack of transparency over content [22].

The authors of the present paper became aware of these considerations while undertaking a scoping review to identify programme theories in preparation for a realist evaluation of the use of Pressure Ulcer Risk Assessment Instruments (PU-RAIs) by nurses in clinical practice. A realist evaluation is a theory driven evaluation concerned with facilitating a deeper understanding of ‘what works, how, for whom, in what circumstances and to what extent’ and involves identifying, testing and refining programme theories [2, 23]. Initially the authors took a conventional empirical approach to literature reviewing and through this learning experience were able to highlight some of the problems encountered and how these were addressed (detailed in

**Table 1:** Summary of methods and results of a Scoping Review of PU-RAI use in clinical practice

**Fig. 1:**
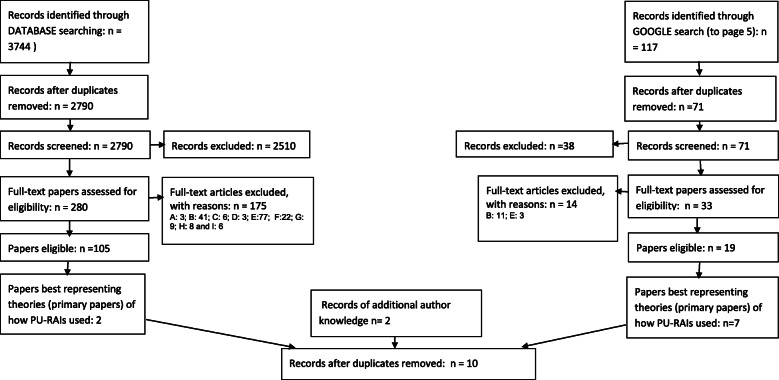
included references found per source and included references available per source

**Introduction** An academic database search was undertaken to identify programme theories associated PU-RAI use. We were looking for a range of publication types and expected to find some grey literature in the academic databases due to their content coverage. This was supplemented with a Google search. **Method** A search strategy incorporating key words relating to risk assessment, pressure ulcers and other areas associated with risk assessment instruments including falls and frailty as well as theories and publication types (systematic review, commentary, opinion piece and editorial) (Appendix 1) was used. Five databases were searched from 1970 to May 2017 (Cochrane Database of Systematic Reviews, Ovid MEDLINE, Ovid HMIC, Ovid Embase, CINAHL EBSCOhost). *Inclusion criteria:* Papers relating to RAIs which describe, review, discuss, critique, provide a theoretical framework or provide stakeholder accounts of use. *Exclusion criteria:* Papers relating to RAIs as a research tool or to assess psychometric properties. *Screening:* After removal of duplicates, the titles, abstracts, and key words of identified papers were screened for relevance by the primary reviewer (SC). Papers considered potentially relevant were obtained in full and reviewed to assess validity and similarity (repetition) of identified programme theories. A 10% random sample of those screened and those potentially relevant were independently screened by a second reviewer (JG). Following identification of papers including relevant theories, the primary papers i.e. those providing clear detail of the possible context and mechanisms through which the intervention/programme is intended to work, were included in the review. *An example of a clearly articulated programme theory associated with this review is detailed below: ‘staff should receive adequate training to ensure they are competent to use the pressure ulcer risk assessment tool. This is especially relevant for novice practitioners who do not have the extensive clinical judgement skills that experienced practitioners have. Therefore risk assessment tools may be especially beneficial for them, particularly when admitting patients in order to prioritise care’ [30]. To summarise one theory that can be drawn from this is that that novice practitioners who do not have extensive clinical judgement skills (Context) may particularly benefit from PU-RAI use (Mechanism) to help them prioritise care (Outcome).* **Results** Despite the database search identifying 2790 potentially relevant papers (after removal of duplicates), only 2 were considered primary papers (Fig. 1). **Subsequent Google Search** Due to the inadequate results of the academic database search a simple Google search of ‘pressure ulcer risk assessment’ and ‘pressure ulcer risk assessment tools’ was undertaken in September 2017. As a large volume of papers were identified and limited resources were available the screening was limited to the first five pages of the Google search. This identified 71 potentially relevant papers (after removal of duplicates) with 6 additional primary papers being of relevance. Two further papers were identified from the researchers’ knowledge (Fig. 1).	

Table 1). Using this example we explore and compare several different (though not all) approaches to literature searching and highlight potential wider implications that could be relevant to others undertaking programme theory scoping reviews, as well as considerations for future apriori methodological studies of literature searching.

### Aim

To compare the performance of a search of academic databases with a simple Google search and inform the development of an optimised search strategy for the identification of programme theories associated with the use of PU-RAIs.

### Objectives

- Compare searches (academic database vs Google vs Google scholar) in identifying primary papers of the scoping review and the resulting screening task (number of references found) from each source.
- Identify reasons for non-retrieval of primary papers in existing searches.
- Test replicability of Google and Google Scholar searches.
- Develop improved search strategies to efficiently capture all primary papers.

## Method

Firstly, we populated a ‘Search Summary Table’ [25] which listed the 10 primary papers and the sources we searched (CINAHL, The Cochrane Library, EMBASE, Google search engine, HMIC, Medline, SC’s personal library) to record:

1. Where our searches had identified each reference in one or more of the sources
2. How many primary references were found per source
3. How many references were retrieved in total from each source
4. The number needed to read (NNR) for each source. NNR is a measure of the precision of a search [26] i.e. the number of titles and abstracts that are screened to identify one relevant reference.
5. In which sources the reference exists and therefore could be found by an ‘optimum search’

The Search Summary Table captures information to determine the minimum set of sources needed to retrieve all primary references for both the academic database search, the Google search and for an ‘optimum search’, that is one which would identify all 10 primary papers. It allows a comparison between the performance of Google and academic database searches in terms of how many of the 10 primary references can be (and were) identified by each.

Secondly, we identified and evaluated instances where references were not found in academic database/Google searches despite their being available in the respective search engine. Understanding the reasons for non-retrieval would help design of new search strategies with improved retrieval of primary papers. We checked terms used for Pressure Ulcers, Risk Assessment Tools and Theories/Systematic review study types within our primary studies to see if we had not used these terms in our original search strategies. The database fields searched (such as title, abstract, index word) was important to determine if we had used the correct terms but in the wrong fields.

Next, we developed and tested improved search strategies for Google and the academic databases to capture all primary papers while retrieving a minimum number of records. We developed an optimised search to identify search methods that could improve precision without losing sensitivity for programme theory reviews. To do this we incorporated our learning of which terms were used in the primary papers and the fields in which they appear in the database record. All search terms used in the original searches were checked to identify if any were redundant (i.e. did not retrieve primary papers). We tested if these search terms could be removed from the search strategy or searched in a more precise manner, e.g. only searching for terms within the title field of database records. We used the NNR calculation to compare the precision i.e. the proportion of results retrieved by the search that are relevant [27] across different sources. We also tested the Google search strategy in Google Scholar to test the impact of searching Google rather than Google Scholar in the original search. Since some literature reviews search Google Scholar rather than Google, a comparison of two could inform the development of the ‘optimum search’.

Finally, in order to understand the replicability of Google and Google Scholar for this particular search, we ran two identical searches on Google and Google Scholar using two PCs based in in the same building, on the same day within a 2 h period. Neither researcher had logged into a Google account on this PC, reducing expected personalisation of results. The first search used the terms ‘Pressure UIcer Risk Assessment’ and the second search used the terms ‘Pressure Ulcer Risk Assessment Tools’. The first five pages of Google and Google Scholar results were downloaded for each search. The replicability test compared results on the first five pages of downloaded records since this was the number downloaded in the original Google search.

## Results

The academic database searches identified 2790 references (after duplicate removal), and the first five pages of the Google search identified 71 references (after duplicate removal). The search results were supplemented with 2 known relevant references from SC’s personal library that had not been identified by either the academic or Google searches. The (NNR) to identify a primary paper from the combined academic databases, Google search and SC’s personal library was 286.3 (2790 refs + 71 refs + 2 refs / 10 primary refs).

### Database comparison

Figure 1 shows the databases where primary papers were found in the searches. It also shows the databases that held these references and could have been located with ‘the optimum search’ but were not necessarily identified with our search. Seven of the ten included references were identified in the Google searches [24, 28–33]. One of these [34] was also found in the database search. Therefore, six references were uniquely found via the Google searches. The NNR for the Google searches was 10.1 references.

**Fig. 2:**
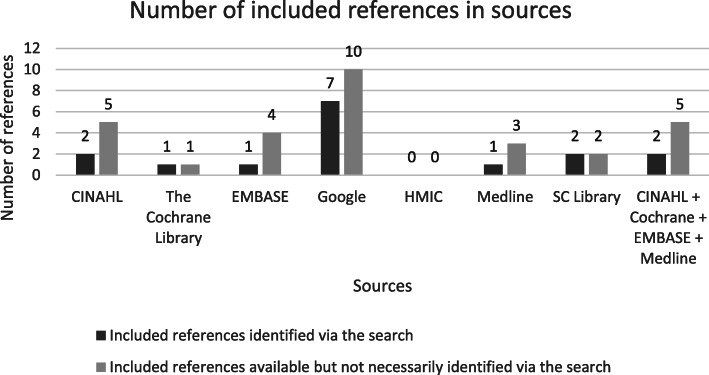
Minimum sources required to find the 10 included references using our search terms (strategy)

Across the academic databases the CINAHL search identified two primary papers. Several database searches were ‘redundant’; HMIC did not identify any relevant references, and The Cochrane Library, EMBASE and Medline searches found two duplicates [34, 35] also found in the CINAHL search. A further two primary papers were identified in SC’s personal library [36, 37]. Using our search strategies, the 10 primary papers could have been identified using three sources; CINAHL, Google and SC’s Personal Library, rather than the seven sources (Fig. 2). The NNR for the academic searches was 1395 while for the combined academic databases and Google search it was 286.3.

### Reasons for non-retrieval in existing searches

**Fig. 3:**
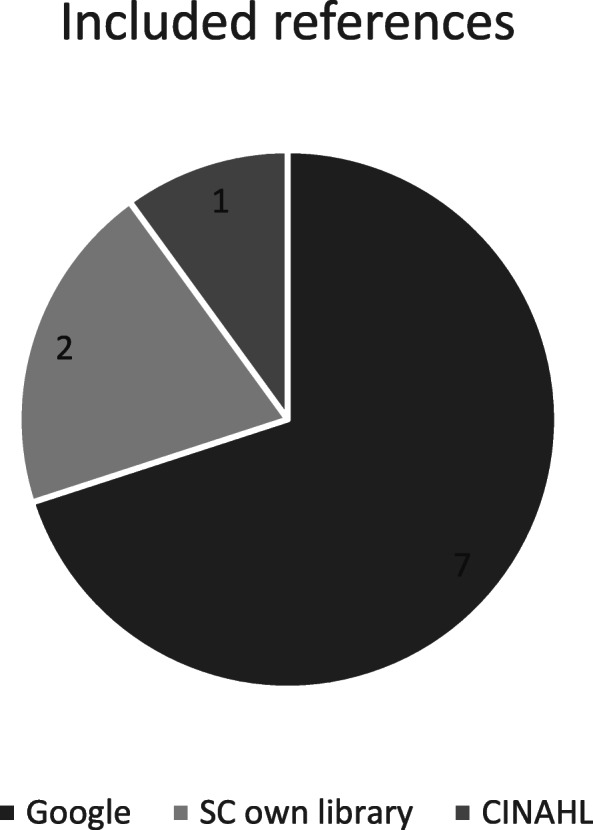
Flow chart of Database and Google search

Figure 3 shows that some primary paper references were available in CINAHL, EMBASE, Medline and Google, but were not identified by the original searches. Table 2 lists the reasons for non-retrieval. All 10 primary references were available in Google, however only 7 appeared in the first five pages of search results from the original two Google searches.

**Table 2:** Retrieval and Non-retrieval of references across databases and reasons for non-retrieval

	Google	Academic databases			
Study reference	Retrieval in Google (non-retrieval reason)	Retrieval in MEDLINE non-retrieval reason)	Retrieval in EMBASE (non-retrieval reason)	Retrieval in CINAHL (non-retrieval reason)	Retrieval in Cochrane (non-retrieval reason)
Bell 2005	✓	X	X	3	X
Chapman 2014	✓	X	X	X	X
Guy 2012	✓	2,3	2, 3	3	X
Green 2017	✓	X	X	X	X
Fletcher 2017	✓	X	2, 3	3	X
Torra i Bou 2006	✓	X	X	X	X
Moore 2014	✓	2	X	✓	✓
Johansen 2014	1	✓	✓	✓	X
Kottner 2010	1	4	4	X	X
EPUAP/ NPUAP 2014	1	X	X	X	X

X: the reference was not stored (or indexed) in the database at time of search

Reasons for non-retrieval when database did contain the reference

1: not within first 5 pages of Google search

2: No age limit indexing

**3**: No terms from theory and publication types search concept

4: In PubMed but not Ovid Medline 1946-present

When the original Google searches were replicated in 2019 the 10 primary references were found within the first 12 pages of Google (i.e. the first 216 records if it is assumed there are 9 references per page on average in Google search results). This indicates that if a larger set of results (216) had been screened from the original search, all 10 primary papers could have been identified with this one source. However, this is an estimate, as we do not have the data for a download of the first 12 pages from the search in 2017.

Five references were not indexed in the academic databases we searched (Table 2). These included a guideline [37], blog item [30], conference abstract [31], book chapter [33] and one journal article [36]. At the time of the original search, the journal reference record was available in PubMed and ‘Ovid Medline In-Process & Other Non-Indexed Citations’ but not the version of Ovid Medline searched (Ovid MEDLINE(R) < 1946 to April Week 32,017>). Of the remaining 5 primary references, CINAHL contained 5 but only retrieved 2, EMBASE contained 4 but only retrieved 1, Ovid Medline (1946-present) contained 4 but only retrieved 1 and The Cochrane Library contained and retrieved 1. The references found in EMBASE, Medline and The Cochrane Library were duplicates of the references found within CINAHL. Three references were not identified by the academic databases searches despite being available in at least one database [28, 29, 32] due to using the search limit ‘Adult’ or ‘Aged’ which the references had not been indexed for [29, 32]. Also search terms (index terms and free text words) used in the search concept for ‘theory and publication types’ were not present in some database indexing records [28, 29, 32].

The optimum search with the lowest NNR of 21.8 was the original Google search (Pressure ulcer risk assessment and Pressure ulcer risk assessment tools). We extended the records screened from five pages (approx. 36 unique records) per Google search to at least 12 pages (approx. 108 unique records) per Google search, in order to retrieve all 10 primary references.

### Replicability of Google and Google scholar searches

The results of the replicability searches in Google where the first five pages were screened by 2 researchers working independently, were similar but not identical. For search (i) ‘Pressure Ulcer Risk Assessment’, 11 results were on the same page for each researcher but in a different order and six references were found by one but not both researchers. For search (ii) ‘Pressure Ulcer Risk Assessment Tool’, 10 results appeared in a different order for each researcher and 22 results were found by one but not both researchers.

Comparison searches were undertaken to test if Google Scholar performed better than Google Searches for identifying our ten primary papers, and to check replicability. The first five pages for each search (76 records in total) were downloaded. The search results from both researchers were exactly the same, indicating strong replicability. However, in both the ‘Pressure Ulcer Risk Assessment’ and the ‘Pressure Ulcer Risk Assessment Tools’ search, only one of the ten primary papers was identified [34]. Google Scholar was inferior to Google in this case study for identifying reports identifying programme theories.

### Optimising the search

In light of our experience in this case study we used a combined but adapted approach to help mitigate publication bias that can arise from only using one source for publications, or in the case of Google searching, an ‘Internet Research Bubble’ [38]. This comprises the existing Google search but extending to page 12 and optimising our academic databases searches by searching only CINAHL, the most relevant database (containing the most included studies (5) with the other databases only containing a subset of these 5). We also optimised the precision of the CINAHL search by searching only in the title field and not the abstract or keywords for the phrases “pressure ulcer” “pressure ulcers” AND “risk assessment”. This would identify the same five available primary references from CINAHL with fewer abstracts needing to be screened. To demonstrate this we ran the specific phrase search in CINAHL (for the same time period of our original search). Search results were limited to studies published up to May 2017, when the original CINAHL search was conducted) and 186 papers were identified with an NNR of 18.6.

The Google and CINAHL optimised searches retrieved 402 references with an NNR of 40.2, whereas the original searches retrieved (2790 + 71 + 2) 2863 references with an NNR 286.3. The researcher would save time in screening 2461 (2863–402) fewer references, and also by not developing complex search strategies and downloading records from four academic databases. If we estimate that it takes on average 2 min to screen each paper with 10% being screened by a second reviewer this would save approximately 90 h of researcher time. This illustrates the large difference in efficiency of the optimised search to the original, though we acknowledge it is impossible to develop such an optimised search when you are developing literature search at the start of the project as you do not yet know the included studies the search needs to find. However, the results indicate some search methods worthy of further testing and research for more efficient identification of programme theory.

## Discussion

Frustration in the results of a search of academic databases for identifying sources of programme theory associated with PU-RAI use (where only 2/10 primary papers found) led to analysis of the search strategy, databases and Google search to understand why. When considering the original database searches for this study we found the same results could have been achieved by searching just CINAHL rather than five databases. This is because pressure ulcers studies are usually conducted by nurses, are most likely to be published in nursing journals and indexed in nursing databases. CINAHL is the only nursing database our institution subscribes to so we could only analyse this. Future research could compare and test the performance of searching other nursing databases e.g. EMCARE, BNI to retrieve programme theory related to PUs. It is acknowledged that the most appropriate database will vary for different subject areas and the scope of the programme theory research questions, e.g. those wider in scope may require consideration of a range of subjects or health specialities, requiring searches of a more multi-disciplinary database, or several databases. Our findings indicate that where the research question relates to a specific speciality covered in depth by a subject database, it could be most efficient to focus searches in the subject database. Further research is required to test if this is the case for other specialities and their subject databases.

The results of the database search highlight inconsistent indexing for age of study population preventing identification of all 5 primary references available. To improve the performance of academic databases in future searches it would be better to identify and then remove ‘child or adolescent’ studies from the search results rather than use the ‘adult’ filter. The original searches would have also found more primary papers if they had not used a ‘theories and publication type’ search concept. It could be argued that the original database search could have been improved and avoided these pitfalls if it had been conducted by an expert searcher rather than an applied health researcher who is less aware of the complexity of searching.

The comparison of the combined academic databases searches (NNR: 1395) with a simple key phrases Google search (NNR: 10.1) found substantially improved precision and sensitivity with the Google search. Given that many primary sources of relevance to programme theory scoping reviews are not indexed in academic databases, they cannot not be retrieved via traditional academic searches. While this may be obvious to those with methodological expertise in the field, for those moving from more traditional research paradigms to realist approaches, this is an important consideration for their search strategies. Indeed, searching grey literature and using supplementary search techniques are considered as important, if not more important than searches of academic literature [39–41], as they can provide contextual richness and reduce bias introduced from peer-reviewed journal articles. Search engines such as Google provide an accessible route to grey literature as well as academic studies that may have been missed in a database search [38, 42]. They provide links to web-based content and reports that can be particularly useful for identifying programme theories and not available in academic databases. For example, a Google search could uncover a blog post or newspaper article discussing opinions of why an intervention works in one setting but not another. This blog data on ideas and assumptions can inform a programme theory and would not be found via an academic database search.

However, the use of search engines such as Google and Google Scholar is controversial in conventional systematic review search methods. They may provide access to large quantities of academic literature and a wide range of ‘grey’ literature of relevance to systematic reviews, but they cannot be searched in the reproducible manner required by conventional systematic reviews [39]. Google search results are personalised in order to display and prioritise results and advertisements it considers most pertinent to the searcher. The exact algorithms used to personalise searches are unknown but are based on factors such as the geographical location of the PC IP address, previous Google searches, and links clicked by the searcher [22]. Personalised search results can introduce bias into systematic reviews by a ‘search bubble effect’ where the tailored search results reflect the searcher’s interests and preferences rather than an impartial representation of websites on the topic searched [38]. It is possible to reduce the personalisation of Google searches and improve their impartiality by using Verbatim search functions, ensuring you are logged out of Google personal accounts, using a private browser window or incognito (Chrome) ([22, 38], and by searching for negative as well as positive views of the intervention under study.

Personalisation of searches may be less of a problem for programme theory reviews, where the aim is to search for each different idea/assumption underlying the intervention under study (rather than trying to comprehensively identify every study with relevant data for a conventional systematic review). Furthermore, the task is to identify *plausible* programme theories, rather than evidence to underpin the validity of those ideas (which occurs in the next stage of a realist review or evaluation). The ranking of results may favour items and websites that are already well known and visited by the researcher, or websites which are geographically close. For programme theory reviews this may be helpful in quickly identifying highly relevant material if the searcher (and the PC they use) has a history of accessing websites and publications on the topic of interest, leading to more relevant items appearing higher up in the ranked Google search results. Unfortunately though, the searcher cannot feel confident they have identified a set of results that is free from bias towards their geographical locations or organisations they are connected with (via Google searches). Search personalisation also remains a problem for developing transparent and replicable results as two searchers using the same search strategy may retrieve different results as noted in our replica Google searches.

Google searches may provide a useful means of informing search terms to be used in academic database searches. Further consideration is needed regarding the impact of cookies, search history and the use various IP addresses in future work. In addition, there is a need to explore and compare alternative search engines to Google such as Bing and DuckDuckGo and different sources of grey literature such as WorldCat, NICE Evidence, OpenGrey in comparison with Google.

Using this case study, we were able to identify an optimum search strategy for programme theories associated with PU-RAI use. Through developing the optimum search strategy, we identified search methods that may help develop more efficient searching for programme theory searches in the future. Searching Google and screening the first 216 (approximately 12 pages) allowed us to find important papers that would otherwise have been missed. If searchers usually screen only the first few pages of Google results, they should consider screening more pages as our case study and guidance for searching grey literature in systematic reviews suggests highly relevant papers are being found in the first 200–300 results [43]. The use of an academic database search (incorporating precise key phrases) in the optimised search recognises that searchers would not feel confident relying on a Google-only search. Searching an academic database provided added transparency and replicability to the searches.

While it is not possible to create such an optimised search when first developing searches for either a systematic or programme theory review, the iterative nature of programme theory development lends itself to testing alternative approaches to identifying relevant papers.

t should also be recognised that the terms used in the database search strategies were not directly comparable with the Google search terms, however it is an example of a real-world search done by a non-expert researcher. Furthermore, we only evaluated the databases and Google for the included studies we had already found. Using other sources may have allowed us to identify additional papers for inclusion but we were unable to evaluate this in the present case study.

What remains unclear is whether our example is typical of other programme theory searches and further research is needed to establish if our results are generalisable to other subject areas. Nevertheless, the findings provide a useful insight which could help researchers undertaking searches for programme theory reviews, rather than conventional systematic reviews. We hope the results of this study can be combined with others in the future to build a more robust evidence base for such searches and inform methodological advancement in the field.

## Conclusion

Limitations of database searches for identifying programme theories associated with PU-RAI use led to consideration of other sources and comparison of academic database and Google searching. The findings indicate the importance of including grey literature sources such as Google in this programme theory search, while acknowledging the need for transparency and replicability. The comparison led to the development a combined and optimised search strategy to efficiently capture primary papers incorporating the extension of the Google search to screen the first 12 pages (rather than the first 5 pages) and a precise search of one large academic database most closely aligned with our topic.

Further research is needed to establish whether these principles are generalisable to other subject areas. This would facilitate further guidance for programme theory searches to enhance practice in the realist field with implications for saving researcher time and therefore resource.

## Supplementary information

**Additional file 1.**

## Data Availability

The datasets during and/or analysed during the current study available from the corresponding author on reasonable request.

## Abbreviations

NNR: Number Needed to Read
PU: Pressure Ulcer
RAI: Risk Assessment Instrument
