# Supplementary material for: Searching for Programme theories for a realist evaluation: a case study comparing an academic database search and a simple Google search
Source: BMC Med Res Methodol. 2020 Aug 26;20:217. doi: 10.1186/s12874-020-01084-x (PMC7450563; doi:10.1186/s12874-020-01084-x)
Supplement: Supplementary file 1 — Additional file 1. [file 12874_2020_1084_MOESM1_ESM.docx]

## Appendix 1 Database searches

Database: Ovid MEDLINE(R) <1946 to April Week 3 2017>

Search Strategy:

--------------------------------------------------------------------------------

1 exp Risk Assessment/ (220024)

2 (risk adj2 assess$).tw. (63600)

3 ((assess$ or predict$) adj10 (tool$ or scale$ or score$ or instrument$ or index$)).tw. (265719)

4 ((braden or waterlow or norton) adj10 (tool$ or scale$ or score$ or instrument$)).tw. (609)

5 exp Skin Ulcer/ (40480)

6 exp Pressure Ulcer/ (11018)

7 skin ulcer$.tw. (2202)

8 (pressure adj1 (ulcer$ or sore$ or wound$ or injur$ or damage$ or lesion$)).tw. (9311)

9 (decubitus or decubital).tw. (4313)

10 ((decubitus or decubital) adj3 (sore$ or ulcer$)).tw. (1783)

11 (bedsore$ or (bed adj1 sore$)).tw. (565)

12 (skin adj3 breakdown$).tw. (728)

13 exp Frail Elderly/ (8821)

14 frail$.tw. (11500)

15 frail older people.tw. (443)

16 age$ associated decline.tw. (507)

17 exp Accidental Falls/ (19571)

18 fall$.tw. (160553)

19 systematic review$.tw. (78090)

20 review$.tw. (1436906)

21 conceptual framework$.tw. (8939)

22 logic model$.tw. (489)

23 theor$.tw. (362487)

24 historical article$.tw. (135)

25 letter$.tw. (66134)

26 comment$.tw. (93586)

27 (discussion adj1 paper).tw. (1019)

28 editorial$.tw. (29654)

29 guideline$.tw. (225085)

30 exp qualitative research/ (33633)

31 qualitative.tw. (141262)

32 mixed method$.tw. (7923)

33 1 or 2 or 3 or 4 (504421)

34 5 or 6 or 7 or 8 or 9 or 10 or 11 or 12 or 13 or 14 or 15 or 16 or 17 or 18 (227622)

35 19 or 20 or 21 or 22 or 23 or 24 or 25 or 26 or 27 or 28 or 29 or 30 or 31 or 32 (2216477)

36 33 and 34 and 35 (2447)

37 limit 36 to (english language and humans and yr="1970 -Current" and "all adult (19 plus years)") (1369)

38 from 37 keep 1-1369 (1369)

Database: HMIC Health Management Information Consortium <1983 - present>

Search Strategy:

--------------------------------------------------------------------------------

1 exp risk assessment/ (1879)

2 (risk adj2 assess$).tw. (1465)

3 ((assess$ or predict$) adj10 (tool$ or scale$ or score$ or instrument$ or index$)).tw. (3922)

4 ((braden or waterlow or norton) adj10 (tool$ or scale$ or score$ or instrument$)).tw. (35)

5 exp skin ulcers/ (134)

6 exp Pressure sores/ (214)

7 skin ulcer.tw. (0)

8 (pressure adj1 (ulcer$ or sore$ or wound$ or injur$ or damage$ or lesion$)).tw. (409)

9 (decubitus or decubital).tw. (23)

10 ((decubitus or decubital) adj3 (sore$ or ulcer$)).tw. (23)

11 (bedsore$ or (bed adj1 sore$)).tw. (18)

12 (skin adj3 breakdown$).tw. (3)

13 exp frail older people/ (190)

14 frail elderly.tw. (339)

15 frail$.tw. (949)

16 age$ associated decline.tw. (3)

17 exp Falling/ (518)

18 accidental fall$.tw. (14)

19 fall$.tw. (3653)

20 systematic review$.tw. (3815)

21 review$.tw. (36752)

22 conceptual framework$.tw. (672)

23 logic model$.tw. (29)

24 theor$.tw. (8720)

25 historical article$.tw. (1)

26 letter$.tw. (3183)

27 comment$.tw. (4953)

28 (discussion adj1 paper).tw. (577)

29 editorial$.tw. (608)

30 guideline$.tw. (9603)

31 exp qualitative research/ (1193)

32 qualitative.tw. (8370)

33 mixed method$.tw. (744)

34 1 or 2 or 3 or 4 (6464)

35 5 or 6 or 7 or 8 or 9 or 10 or 11 or 12 or 13 or 14 or 15 or 16 or 17 or 18 or 19 (5228)

36 20 or 21 or 22 or 23 or 24 or 25 or 26 or 27 or 28 or 29 or 30 or 31 or 32 or 33 (65556)

37 34 and 35 and 36 (74)

38 limit 37 to (yr="1970 -Current" and english) (64)

Database: Embase <1996 to 2017 Week 17>

Search Strategy:

--------------------------------------------------------------------------------

1 exp risk assessment/ (381819)

2 (risk adj2 assess$).tw. (87298)

3 ((assess$ or predict$) adj10 (tool$ or scale$ or score$ or instrument$ or index$)).tw. (412064)

4 ((braden or waterlow or norton) adj10 (tool$ or scale$ or score$ or instrument$)).tw. (730)

5 exp skin ulcer/ (46884)

6 exp decubitus/ (13579)

7 skin ulcer$.tw. (2641)

8 (pressure adj1 (ulcer$ or sore$ or wound$ or injur$ or damage$ or lesion$)).tw. (10347)

9 (decubitus or decubital).tw. (4184)

10 ((decubitus or decubital) adj3 (sore$ or ulcer$)).tw. (1149)

11 (bedsore$ or (bed adj1 sore$)).tw. (613)

12 (skin adj3 breakdown$).tw. (849)

13 exp frail elderly/ (7617)

14 frail$.tw. (17842)

15 frail older people.tw. (592)

16 age$ associated decline.tw. (504)

17 exp falling/ (29475)

18 falls.tw. (39172)

19 systematic review$.tw. (116776)

20 review$.tw. (1735497)

21 conceptual framework$.tw. (9412)

22 logic model$.tw. (667)

23 theor$.tw. (377765)

24 historical article$.tw. (149)

25 letter$.tw. (109634)

26 comment$.tw. (133389)

27 editorial$.tw. (65543)

28 (discussion adj2 paper).tw. (1052)

29 guideline$.tw. (350741)

30 exp qualitative research/ (42365)

31 mixed methods.tw. (8367)

32 qualitative.tw. (167193)

33 1 or 2 or 3 or 4 (800960)

34 5 or 6 or 7 or 8 or 9 or 10 or 11 or 12 or 13 or 14 or 15 or 16 or 17 or 18 (126902)

35 19 or 20 or 21 or 22 or 23 or 24 or 25 or 26 or 27 or 28 or 29 or 30 or 31 or 32 (2729753)

36 33 and 34 and 35 (3092)

37 limit 36 to (human and english and yr="1970 -Current" and adult <18 to 64 years>) (583)

38 limit 36 to (human and english language and yr="1970 -Current" and aged <65+ years>) (978)

39 37 or 38 (1241)

Database: Cochrane Database of Systematic Reviews (Wiley)

Date Run: 03/05/17 15:28:16.444

#1 MeSH descriptor: [Risk Assessment] this term only 9256

#2 MeSH descriptor: [Skin Ulcer] this term only 159

#3 MeSH descriptor: [Pressure Ulcer] this term only 674

#4 MeSH descriptor: [Frail Elderly] this term only 636

#5 MeSH descriptor: [Accidental Falls] this term only 1314

#6 risk assessment:ti,ab,kw (Word variations have been searched) 35222

#7 assess or predict next (tool* or score* or scale* or instrument* or index*) 100913

#8 braden or waterlow or Norton next (tool* or score* or scale* or instrument* or index*) 190

#9 decubitus or decubital:ti,ab,kw (Word variations have been searched) 706

#10 decubitus or decubital:ti,ab,kw and sore* or ulcer* (Word variations have been searched) 291

#11 bedsore:ti,ab,kw (Word variations have been searched) 31

#12 skin:ti,ab,kw and breakdown (Word variations have been searched) 206

#13 frail:ti,ab,kw (Word variations have been searched) 1387

#14 frail older people:ti,ab,kw (Word variations have been searched) 267

#15 age* associated decline:ti,ab,kw (Word variations have been searched) 3671

#16 fall*:ti,ab,kw (Word variations have been searched) 13890

#17 #1 or #6 or #7 or #8 129347

#18 #2 or #3 or #4 or #5 or #9 or #10 or #11 or #12 or #13 or #14 or #15 or #16 20131

#19 #17 and #18 Publication Year from 1970 552

Database: CINAHL (EBSCOhost)

Date run: Tuesday, May 02, 2017 10:20:12 AM

S1 (MH "Risk Assessment") 41,603

S2 TI Risk Assessment OR AB Risk Assessment 11,294

S3 TI (risk n2 assess*) OR AB (risk n2 assess*) 15,533

S4 TI ( ((assess* or predict*) n10 (tool* or scale* or score* or instrument* or index*)) ) OR AB ( ((assess* or predict*) n10 (tool* or scale* or score* or instrument* or index*)) ) 66,170

S5 TI ( (braden or waterlow or norton) n10 (tool* or scale* or score* or instrument*) ) OR AB ( (braden or waterlow or norton) n10 (tool* or scale* or score* or instrument*) ) 646

S6 S1 OR S2 OR S3 OR S4 OR S5 114,026

S7 (MH "Skin Ulcer+") 19,557

S8 TI "skin ulcer*" OR AB "skin ulcer*" 241

S9 (MH "Pressure Ulcer+") 9,674

S10 TI ( (pressure n1 (ulcer* or sore* or wound* or injur* or damage* or lesion*) ) OR AB ( (pressure n1 (ulcer* or sore* or wound* or injur* or damage* or lesion*) ) 8,171

S11 TI ( decubitus or decubital ) OR AB ( decubitus or decubital ) 530

S12 TI ( (bedsore* or (bed n1 sore*) ) OR AB ( (bedsore* or (bed n1 sore*) ) 163

S13 TI skin n3 breakdown* OR AB skin n3 breakdown* 435

S14 MH "Frail Elderly" 4,044

S15 TI frail* OR AB frail* 5,227

S16 TI "age* associated decline" OR AB "age* associated decline" 40

S17 (MH "Accidental Falls") 12,436

S18 TI fall* OR AB fall* 26,200

S19 S7 OR S8 OR S9 OR S10 OR S11 OR S12 OR S13 OR S14 OR S15 OR S16 OR S17 OR S18 58,171

S20 MH "Systematic Review" 28,343

S21 TI review* OR AB review* 264,678

S22 MH "Scoping Review" 16

S23 (MH "Conceptual Framework") 28,475

S24 TI "conceptual framework*" OR AB "conceptual framework*" 4,409

S25 TI "logic model*" OR AB "logic model*" 205

S26 MH "Models, Theoretical" 25,500

S27 TI theor* OR AB theor* 69,110

S28 TI "historical article*" AND AB "historical article*" 0

S29 TI letter* OR AB letter* 25,631

S30 TI comment* OR AB comment* 48,880

S31 TI discussion n1 paper* OR AB discussion n1 paper* 678

S32 MH "Edit and Review" 1,863

S33 TI editorial* OR AB editorial* 25,156

S34 (MH "Practice Guidelines" 39,287

S35 TI guideline* OR AB guideline* 65,645

S36 MH "Qualitative Studies" 60,743

S37 TI "qualitative research" OR AB "qualitative research" 6,433

S38 TI "mixed method*" OR AB "mixed method*" 5,283

S39 S20 OR S21 OR S22 OR S23 OR S24 OR S25 OR S26 OR S27 OR S28 OR S29 OR S30 OR S31 OR S32 OR S33 OR S34 OR S35 OR S36 OR S37 OR S38 570,283

S40 S6 AND S19 AND S39 518
